# Haloperidol induces pharmacoepigenetic response by modulating miRNA expression, global DNA methylation and expression profiles of methylation maintenance genes and genes involved in neurotransmission in neuronal cells

**DOI:** 10.1371/journal.pone.0184209

**Published:** 2017-09-08

**Authors:** Babu Swathy, Moinak Banerjee

**Affiliations:** Human Molecular Genetics Laboratory, Rajiv Gandhi Centre for Biotechnology, Trivandrum, Kerala, India; Wayne State University, UNITED STATES

## Abstract

**Introduction:**

Haloperidol has been extensively used in various psychiatric conditions. It has also been reported to induce severe side effects. We aimed to evaluate whether haloperidol can influence host methylome, and if so what are the possible mechanisms for it in neuronal cells. Impact on host methylome and miRNAs can have wide spread alterations in gene expression, which might possibly help in understanding how haloperidol may impact treatment response or induce side effects.

**Methods:**

SK-N-SH, a neuroblasoma cell line was treated with haloperidol at 10μm concentration for 24 hours and global DNA methylation was evaluated. Methylation at global level is maintained by methylation maintenance machinery and certain miRNAs. Therefore, the expression of methylation maintenance genes and their putative miRNA expression profiles were assessed. These global methylation alterations could result in gene expression changes. Therefore genes expressions for neurotransmitter receptors, regulators, ion channels and transporters were determined. Subsequently, we were also keen to identify a strong candidate miRNA based on biological and in-silico approach which can reflect on the pharmacoepigenetic trait of haloperidol and can also target the altered neuroscience panel of genes used in the study.

**Results:**

Haloperidol induced increase in global DNA methylation which was found to be associated with corresponding increase in expression of various epigenetic modifiers that include *DNMT1*, *DNMT3A*, *DNMT3B* and *MBD2*. The expression of miR-29b that is known to putatively regulate the global methylation by modulating the expression of epigenetic modifiers was observed to be down regulated by haloperidol. In addition to miR-29b, miR-22 was also found to be downregulated by haloperidol treatment. Both these miRNA are known to putatively target several genes associated with various epigenetic modifiers, pharmacogenes and neurotransmission. Interestingly some of these putative target genes involved in neurotransmission were observed to be upregulated while CHRM2 gene expression was down regulated.

**Conclusions:**

Haloperidol can influence methylation traits thereby inducing a pharmacoepigenomic response, which seems to be regulated by DNMTs and their putative miRNA expression. Increased methylation seems to influence CHRM2 gene expression while microRNA could influence neurotransmission, pharmacogene expression and methylation events. Altered expression of various therapeutically relevant genes and miRNA expression, could account for their role in therapeutic response or side effects.

## Introduction

Antipsychotic medications are considered as first line treatment for schizophrenia and related psychotic disorder. It has been more than half a century that antipsychotic drugs have reached clinical settings, but still there are many challenges in the antipsychotic drug therapy. Among antipsychotic medications, haloperidol is the first typical antipsychotic drug widely used for treating the positive and negative symptoms associated with schizophrenia. It exerts its antipsychotic effect primarily through the antagonism of dopamine receptors in the brain. Despite its drug specific effects, haloperidol is associated with various adverse effects including extra pyramidal symptoms.

Several mechanisms are known to be involved in the antipsychotic activity and related side effects. Studies have shown that haloperidol treatment is associated with changes in gene expression involved in neurotransmission, neural plasticity, oxidative stress, signal transduction, ionic homeostasis and metabolism [1–3]. Haloperidol also alters gene expression patterns that results in serious side effects [4–7]. It is plausible that the altered gene expression patterns could be mediated by epigenetic mechanisms. Therefore, understanding the epigenetic alterations induced by haloperidol could help us understand the potential pharmacoepigenetic impact of haloperidol on therapeutic response and side effects. The postulated epigenetic effect could be either at global or gene specific level induced by DNA methylation, histone modifications or microRNA (miRNA) expression. Large numbers of studies have identified the role of epigenetics in Schizophrenia and emerging reports also indicate that antipsychotics can also influence the host epigenome [8]. In a conventional clinical setup it is difficult to evaluate the effect of individual antipsychotic drug on host epigenome. This prompted us to study the impact of widely used antipsychotic haloperidol on host epigenome.

In the present study, we propose to investigate whether haloperidol can alter global DNA methylation events in a biologically relevant concentration, under an in-vitro neuronal cell culture model, and if so, what mediates these global methylation alterations. The impact of these global methylation events and the role of miRNAs in influencing the expression of neuroscience panel of genes and pharmacologically relevant genes will also be investigated. Observations from the study will help in understanding the crosstalk between epigenetics and pharmacoepigentic response, which is difficult in a clinical setting.

## Methods

### Cell culturing

Human neuroblastoma cell line, SK-N-SH (ATCC(®) HTB-11™) was maintained in Dulbecco's modification of Eagle's medium (DMEM; Gibco) supplemented with 10% fetal bovine serum (Gibco), 1X antibiotic antimycotic solution (Invitrogen) in a 37°C humidified incubator with 5% CO2. Cells were seeded at a density of 3 × 10^5^ cells/well in 6 well plates and each 6 well plate was used for each treatment concentration and timing. Each experimental protocol was carried out in triplicates. All experiments were performed between passages 5 and 12. Short Tandem Repeat (STR) profiling of SK-N-SH cell line was carried out using AmpFLSTR Identifiler amplification kit (Applied Biosystems). Cell line authentication confirmed the identity of the cell line as described in American Tissue Culture Collection (ATCC).

### Drug treatment

Haloperidol (HLP) procured from Sigma Chemical Company was dissolved in dimethylsulfoxide (DMSO). For control samples, fresh medium (including DMEM or DMSO as vehicle) was added. The final concentration of DMSO in the medium was less than 0.25%. Pilot experiments were carried out using various concentrations of HLP (0μM, 1μM, 10μM and 25μM) for different time intervals (6hr, 12hr, 18hr and 24hr). In these experiments most significant alterations were observed at 10μM HLP following 24hr treatment and this concentration was used for subsequent downstream experiments.

### DNA extraction and quantification of global DNA methylation

DNA was prepared from cell lines using Pure Link Genomic DNA Mini Kit (Invitrogen), according to the manufacturer's instructions. Quality and quantity of DNA was measured using NanoDrop 1000 spectrophotometer (Thermo Scientific). Quantification of global DNA methylation (5-mC) was done using colorimetric assay, MethylFlash™ Methylated DNA 5-mC Quantification Kit (Epigentek) following the manufacturer’s instructions. MethylFlash™ kit employs methylation controls including positive and negative control. Relative quantification of DNA methylation was calculated using the following formula.

$$5mC=\frac{\left((sample\;OD-Negative\;Control\;OD)/S\right)}{(Positive\;Control\;OD-Negative\;Control\;OD)X\;2*/P}X\;100$$

S is the amount of input sample DNA in ng. P is the amount of input positive control in ng. 2* is a factor to normalize 5-mC in the positive control to 100%, as the positive control contains only 50% of 5-mC.

### RNA extraction, reverse transcription and real-time PCR analysis

Total RNA extraction was performed from cell lines using the TRIzol reagent (Invitrogen), according to manufacturer's instructions. The total RNA was quantified using Nanodrop ND-1000 spectrophotometer and its quality was assessed by running in 1.2% agarose gel.1μg of total RNA was used for cDNA synthesis using High Capacity Reverse Transcriptase kit (Applied Biosystems Part No:4368814) as per the manufacturer’s instruction. The expression of epigenetic genes (*DNMT1*, *DNMT3A*, *DNMT3B*,*MBD2*) were analysed using real time quantification of cDNA using either Taqman gene expression assay (Applied Biosystems) or SYBR Green based assays (Applied Biosystems). ß-actin (ACTB) was used as the endogenous control. TaqMan assays were purchased from Applied Biosystems. (DNMT1-Hs00945875_m1;MBD2- Hs00969366_m1 and ACTB- Hs01060665_g1).The SYBR Green primers were designed using QuantPrime and the sequences are as *follows*: *DNMT3A*,5’-ACATCTCGCGATTTCTCGAGTCC-3’ and 5’-GTGCAGCTGACACTTCTTTGGC-3’;*DNMT3B*,5’-ACAACAAGAGCAGCCTGGAAGATG-3’and 5’-AAAGAGAGGGTGGAAGGACACG3’; *ACTB*,5’-AATCTGGCACCACACCTTCTA-3’ and 5’-ATAGCACAGCCTGGATAGCAA-3’. In addition to these epigenetic genes we also profiled a panel of Human Neurotransmitter Receptors and Regulators (PAHS-060A-2) and Human Neuroscience Ion Channels & Transporters(PAHS-036A-2) using a 96-well PCR-array platform of RT2 Profiler PCR Arrays procured from Qiagen. All real time PCR data were captured using Sequence Detector Software (SDS version 2.4; Applied Biosystems). The real-time PCRs were run in triplicate. Relative quantification of gene expression levels of the target genes were determined by the 2^-ΔΔCt^ method. For PCR arrays, the data was normalized to the average of two housekeeping genes, *ACTB* and Ribosomal protein Ll3a, which showed most consistent Ct(cycle threshold) values. All real time PCRs was performed with reference to the Minimum Information for Publication of Quantitative Real-Time PCR Experiments guidelines shown in S1 Table

### MicroRNA isolation, reverse transcription and real-time PCR analysis

MicroRNA was extracted from cells using mirVana™ miRNA Isolation kit (Ambion Ltd.). High-Capacity cDNA Archive Kit (Applied Biosystems, Part No:4322171,) was used for reverse transcription, following manufacturer's protocol. For the Real-time PCR step, amplification was carried out using Applied Biosystems TaqMan® microRNA (miRNA) assays (designed for mature miRNA quantification using Applied Biosystems Real Time PCR instruments). Following miRNA assays for hsa-miR-29b-3p Assay ID: 000413 and hsa-miR-22 Assay ID: 000398 (Applied Biosystems) were used. The real-time PCRs were run in triplicate. SnoU6 (Assay ID: 001973, Applied Biosystems) was included as an endogenous control. The normalized expression values were represented.

### Statistical analysis

Data are presented as means ± SEM. The statistical significance of differences among groups was evaluated by Student's t-test. A P- value of <0.05 was considered statistically significant. All these analyses were implemented using the Graph Pad Prism 5.01.

## Results

We have performed global DNA methylation studies in various concentrations (0μM, 1μM, 10μM and 25μM) for different time intervals (6hr, 12hr, 18hr and 24hr) (S2 Table). Most significant alterations were observed at 10μM HLP following 24hr treatment; therefore, we have restricted subsequent gene expression studies to drug concentration of 10μM HLP and time interval of 24hr treatment.

### Antipsychotic drugs induce global DNA methylation changes in SK-N-SH

**Fig 1:**
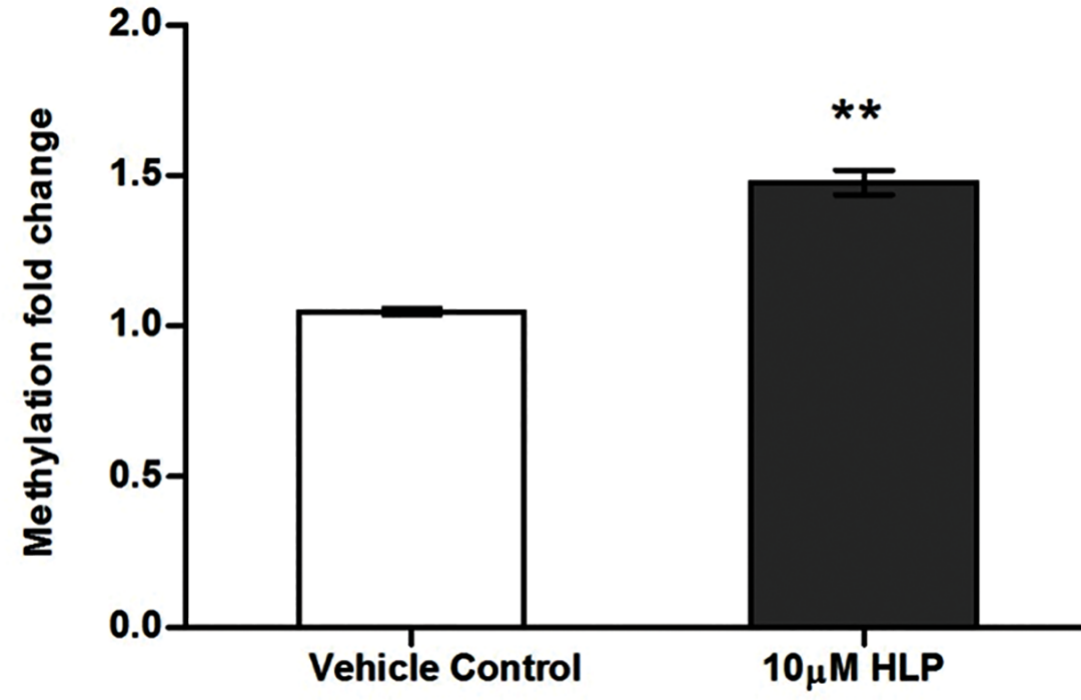
Effect of HLP on global DNA methylation in SK-N-SH cells.

The effect of haloperidol on global DNA methylation was assessed in SK-N-SH following 10 μM HLP treatment for 24 hrs. The average % of methylation in control sample was 2.68% and that in HLP treated sample was 3.96% and hence the % difference is 47.76. Thus the global 5-methylcytosine levels increased by 1.47 fold (P = 0.001) following treatment of 10μM of HLP for 24hr compared to control [Fig 1].

### Haloperidol induces increased expression of epigenetic genes

**Fig 2:**
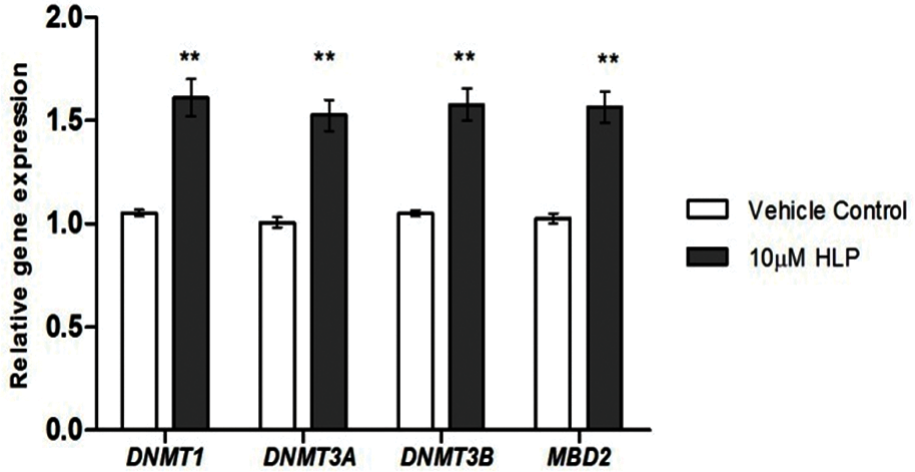
Effect of HLP on gene expression of epigenetic genes in SK-N-SH cells.

In order to determine whether the increase in global DNA methylation was due to the altered expression of various epigenetic modifiers, we measured the gene expression status of various epigenetic genes involved in DNA methylation process. Gene expression of DNA methyltransferases (*DNMT1*, *DNMT3A*, *DNMT3B)* and MethylCpG binding protein *(MBD2)* were determined following treatment of 10μM HLP for 24hr using quantitative real time PCR. We observed increase in all DNMT and MBD2 expression post antipsychotic treatment. *DNMT1* expression increased by 1.6 fold (P = 0.002) whereas *DNMT3A*, *DNMT3B* and *MBD2* increased by 1.5 fold (*DNMT3A*, P = 0.002; *DNMT3B* and *MBD2*,P = 0.001) [Fig 2].

### Haloperidol down regulates miR-29b expression

MicroRNA expression can regulate the DNA methylation by targeting the DNA methylation machinery. We next wanted to examine whether any miRNA could mediate the haloperidol induced increase in global methylation or expression of epigenetic genes. miR-29b is known to regulate DNA methylation by targeting various epigenetic modifiers. Computational prediction of target genes of miR-29b using miRWalk database (zmf.umm.uni-heidelberg.de/apps/zmf/mirwalk2/) identified *DNMT1*, *DNMT3A*, *DNMT3B* and *MBD2* as putative targets (S3 Table). miRNet database (www.mirnet.ca.) provided a miRNA-target interaction network presenting various targets of DNMTs [S1 Fig].

**Fig 3:**
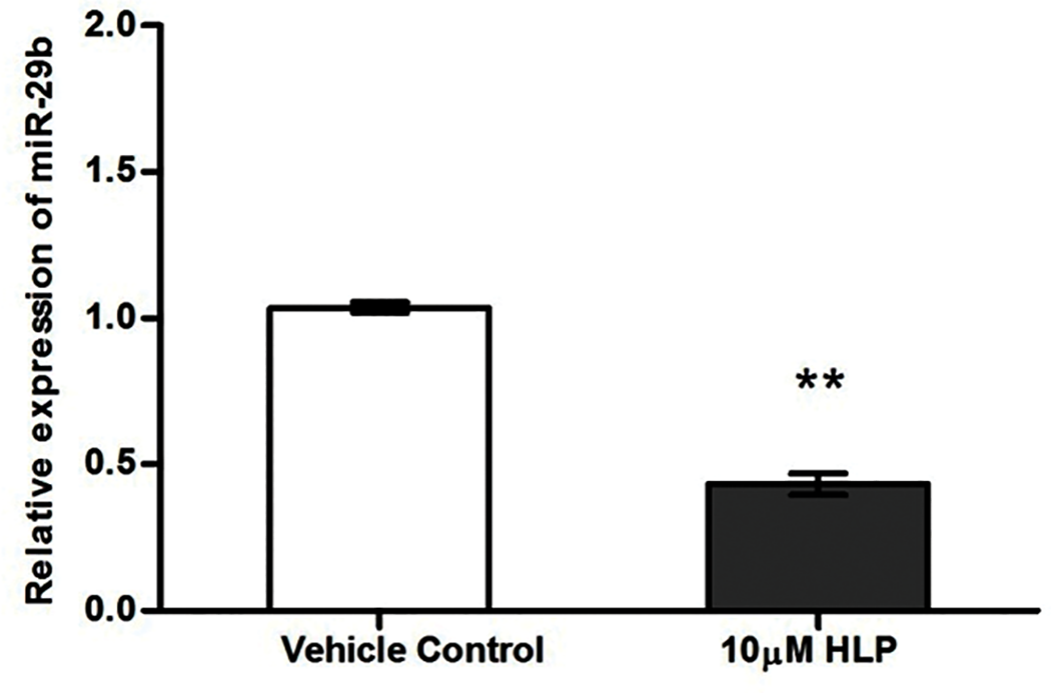
Effect of HLP on miR-29b expression in SK-N-SH cells.

To test the possibility that miR-29b regulates these genes, we assessed the expression of miR-29b following haloperidol treatment using quantitative real time PCR. The expression of miR-29b was significantly down regulated by 10μM HLP by 2.3 fold (P = 0.0005) [Fig 3]. The decreased expression of miR-29b could be correlated with the increased expression of epigenetic modifiers which in turn leads to increased DNA methylation.

### Haloperidol induce alterations in the expression of genes involved in neurotransmission

Haloperidol induced modulation of epigenome could reflect in the gene expression status of various genes involved in haloperidol drug function. Our next objective was to determine the effect of haloperidol in altering the expression of various genes involved in neurotransmission. To study this aspect, we used two real time based gene expression arrays: Neurotransmitter Receptors and Regulators and Human Neuroscience Ion Channels & Transporters. In the human neurotransmitter receptors and regulators array, we observed upregulation of 4 genes and downregulation of 1 gene with 10μM HLP treatment [Table 1]. In the human neuroscience ion channels and transporters array, 3 genes were up regulated with 10μM HLP treatment. The fold change in expression is relative to control and corresponding P values are represented in the Table 1.

**Table 1:** Effect of HLP on expression of neurotransmitter receptors, regulators, neuroscience related ion channels and transporters.

Array Panel	Gene	Description	Fold change	P value
Neurotransmitter Receptors and regulators	TSPO	Translocator Protein	1.35	0.007
Neurotransmitter Receptors and regulators	CHRM2	Cholinergic receptor muscarinic 2	0.80	0.008
Neurotransmitter Receptors and regulators	CHRNA5	Cholinergic receptor, nicotinic, alpha 5	3.63	0.002
Neurotransmitter Receptors and regulators	DRD1	Dopamine receptor D1	3.87	0.002
Neurotransmitter Receptors and regulators	GCH1	GTP-cyclohydrolase 1	1.83	0.004
Ion channels and transporters	ATP1A1	ATPase, Na+/K+ transporting, alpha 1 polypeptide	2.19	0.003
Ion channels and transporters	ATP1B1	ATPase, Na+/K+ transporting, beta 1 polypeptide	1.24	0.010
Ion channels and transporters	ATP2A1	ATPase, Ca++ transporting, cardiac muscle, fast twitch 1	1.17	0.013

### Haloperidol treatment down regulates miR-22 expression

**Fig 4:**
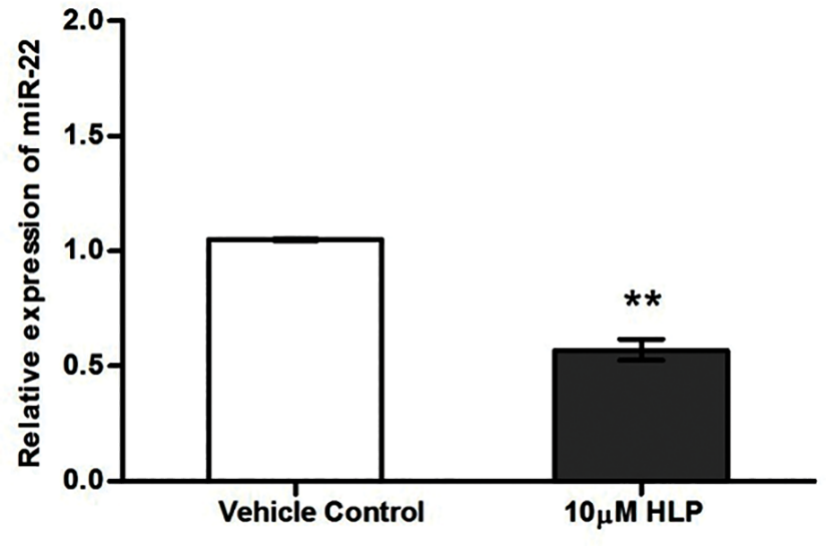
Effect of HLP on miR-22 expression in SK-N-SH cells.

We wanted to identify a microRNA that could target maximum number of pharmacogenetic genes and that could also target the neuroscience panel of genes that are significantly altered in the study. To resolve this we used a combination of in-silico and validation tool. Selection of miRNAs associated with haloperidol was obtained from the Pharmaco-miR, the miRNA pharmacogenomics database [www.Pharmaco-miR.org], which showed 137 miRNAs. The lists of miRNAs are given in S4 Table (. The genes associated with haloperidol drug function were obtained from the Drug bank database (www.drugbank.ca/) and literature survey S5 Table). The interaction of candidate miRNAs with genes associated with haloperidol drug function was predicted by miRWalk S6 Table. (MicroRNAs including miR-150, miR-22, miR-300, miR-520c-3p and miR-520e targets maximum number of genes associated with haloperidol drug function. Among all these microRNAs evidence for validation for haloperidol action was reported only for miR-22. Therefore, expression status of miR-22 was carried out, which was selected based on the in-silico tools and previous validation reports on its implications in antipsychotic mechanism of action. We assessed the expression of miR-22 following 10μM HLP treatment for 24 hours. [Fig 4] The expression of miR-22 was significantly down regulated by 10μM HLP by 1.75 fold (P = 0.002). Interestingly in-silico prediction using miRwalk identified that miR-22 and miR-29b could also target *TSPO*, *DRD1*, *GCH1*, *ATP1A1* and *ATP1B1* S7 Table. The increased expression of these genes might be regulated by reduced expression of these miRNAs.

## Discussion

The present study extensively explores the role of haloperidol in modulating the epigenome and gene expression status of epigenetic modifiers and neurotransmitter related genes that are likely to be therapeutically relevant. DNA methylation is a key epigenetic mechanism that controls gene expression therefore, we first investigated whether haloperidol, a typical antipsychotic drug can influence global DNA methylation. Our results have shown that haloperidol treatment increased the global DNA methylation at 5-methylcytosine level. This observation is interesting as inconsistencies in global DNA methylation have been reported in Schizophrenia patients selected from conventional treatment background. Therefore, the inconsistencies in global methylation could be due to background medication effects. Our next focus was to determine whether the increase in global DNA methylation was due to the increased gene expression of any of the methylation maintenance genes such as DNA methyltransferase (DNMT) enzymes- *DNMT1* (maintenance methyltransferase), *DNMT3A* and *DNMT3B* (de novo methyltransferases) and various methyl-CpG binding proteins such as *MBD2*, *MBD1* and *MECP2* that can bind specifically to methylated DNA thus aiding methylation. Interestingly, with haloperidol treatment we observed an increase in expression of various DNA methyltransferases (*DNMT1*, *DNMT3A* and *DNMT3B)* and methyl CpG binding protein (*MBD2*). Thus the increase in 5-methylcytosine level could be explained by the increase in expression of these epigenetic modifiers. It is possible that microRNAs can also influence DNA methylation by targeting various epigenetic genes. Various miRNA target prediction programs have identified that the genes involved in the methylation maintenance machinery that could influence global methylation such as DNMTs are the putative targets of miR-29b identified based on in-silico and validation tools. A previous study has also reported that miR-29b promotes global DNA hypermethylation by targeting directly DNMT3A and 3B and indirectly DNMT1 in acute myeloid leukemia[9]. In similar lines the present study also clearly demonstrates that haloperidol treatment down regulates miR-29b expression which might explain the increase in DNMT expression, resulting in global DNA hypermethylation. This suggests that haloperidol induces epigenetic alterations, which seems to be are modulated by miRNA expression.

Drug induced epigenetic modifications can also influence the pharmacokinetic and pharmacodynamic properties, resulting in altered therapeutic response. Therapeutic relevance of haloperidol was monitored by considering entire range of neurotransmitter receptors, neurotransmitter regulators, Ion channels and various ion-transporter genes. Our gene expression analysis revealed that various neurotransmitter receptors, regulators and transporters were differentially expressed after haloperidol treatment. Among these differentially expressed genes the downregulation of CHRM2 seems to be the biologically relevant outcome of our study based on our methylation observations. This observation is interesting, as decreased cortical CHRM2 a Muscarinic Acetylcholine Receptor, has been reported to define a subgroup of subjects with schizophrenia.[10] Our observation suggests that it could be an effect of haloperidol treatment which is further supported with the evidence that increased antipsychotic dosage can influence autonomic nervous system activity through CHRM2.[11] Some of the genes in the neurotransmitter receptors, regulators, neuroscience related ion channels and transporters were found to be increased in expression which was contrast to our global methylation observations, which needs to be investigated further. This contrast has been reported by other investigators too showing global hypomethylation and gene specific hypermethylation in Schizophrenia. [8] One would speculate that haloperidol might induce gene specific hypomethylation in neuroscience panel of genes resulting in increased gene expression in garnering treatment response. Interestingly in-silico prediction identified that miR-22 and miR-29b could also target *TSPO*, *DRD1*, *GCH1*, *ATP1A1* and *ATP1B1*. Therefore the increased expression of these genes might be regulated by reduced expression of these miRNAs. However, this needs further validation.Some of the genes represented in the arrays could not be used for analysis possibly due to lack of expression in SK-N-SH. This observation was further verified from the database on transcriptional portrait of SK-N-SH cells [12], supporting our observation on lack of expression.

Differential expression in genes of pharmacogenetic interest and the role of miRNAs in influencing global methylation stimulated us to identify most potential miRNAs that could have a role in modulating a pharmacogenetic response. To resolve this issue we used a combination of in-silico and validation tool. In-silico tool identified five most potential miRNAs and among these miRNAs, validated observation was available for only miR-22. This prompted us to experimentally verify the expression of miR-22 post haloperidol treatment. Haloperidol treatment induced significant down regulation of miR-22. Almost a similar observation on down regulation of miR-22 in haloperidol-treated mice has been reported earlier [13]. This clearly suggests that miR-22 can negatively regulate the expression of various therapeutically important genes that could be involved in haloperidol drug action. Evaluation of these observations in clinical setup might help us distinguish the role of these miRNA in monitoring therapeutic response and side effects.

A few studies have previously reported epigenetic mode of action of antipsychotic drugs including haloperidol. Haloperidol treatment was shown to be associated with higher global DNA methylation in schizophrenia patients [14]. Haloperidol is also reported to induce sex and tissue specific changes in global DNA methylation in rat models [15]. Haloperidol can also increase H3 phospho-acetylation (H3pS10-acK14) in whole chromatin from striatal extracts in in-vivo animal model [16]. A couple of studies have reported miRNAs alteration with haloperidol treatment. Three miRNAs, mir-128a, mir-128b and mir-199a showed higher expression in haloperidol-treated rats [17]. miR-22 and miR-434-5p was reported to be down regulated with haloperidol treatment in mice[13]. In addition to haloperidol other antipsychotic drugs are also reported to modulate a pharmacoepigenomic response. Olanzapine induces methylation changes in genes involved in dopamine pathway including DRD1, DRD2, DRD5, COMT, SLC18A2 and DDC8 in rat model. [18] In a recent genome wide methyation study, olanzapine is reported to induce widespread tissue-specific methylation changes in rats.[19] Same study has reported that olanzapine treatment increases the methylation of miRNA gene Mir125b-1 in cerebellum, thus altering expression of corresponding miRNA. Risperidone is also reported to modulate the expression levels of miR-346, miR-365 and miR-520c-3p in serum [20]. In the present study we chose to use SK-N-SH, a cell line of neuronal origin as neuronal cells are the major targets of antipsychotic drugs. The choice of concentration of haloperidol for treatment was restricted to 10μM HLP as it mimics the acute phase concentration in central nervous system and is clinically relevant, as the haloperidol concentration reaching brain is 10–30 times higher than the serum concentration [21]. The experimental duration of 24 hours with10μM HLP under in-vitro condition, is expected to simulate long term effects of the drugs under in-vivo conditions in humans.

In conclusion, our data on in-vitro human neuronal cell model indicates that haloperidol can induce epigenetic effects by influencing global DNA methylation. The global DNA methylation event seems to be modulated by miRNA mediated DNMT gene expression. DNA methylation and miRNA expression may partially modulate the neurotransmitter receptors, regulators, neuroscience related ion channels and transporters in mediating a pharmacogenetic response. These observations will help in furthering the knowledge in resolving the harmful and beneficial effects of haloperidol treatment. In addition it will also aid in distinguishing the pharmacoepigenetic response from epigenetic response in pathogenesis of schizophrenia.

## Supporting information

S1 Fig Network-based visual analysis of *DNMT1*, *DNMT3A* and *DNMT3B* and their miRNA targets. (EPS)

S1 Table Minimum Information for Publication of Quantitative Real-Time PCR Experiments guidelines followed in the study. (XLS)

S2 Table The table presents the mean fold changes in global DNA methylation relative to control at different haloperidol treatment concentrations and time intervals. (DOCX)

S3 Table Putative targets of miR-29b predicted by different algorithms in miRWalk. (0 = not predicted as target by algorithm,1 = predicted as target by algorithm).The interaction of miR-29b with *DNMT1*,*DNMT3A*,*DNMT3B* and *MBD2* was predicted by 2,7,7 and 4 algorithms respectively.
(DOCX)

S4 Table The list of miRNAs extracted from miRNA pharmacogenomics database which showed 137 miRNAs. (XLSX)

S5 Table Genes associated with haloperidol drug function were obtained from the Drug bank database and literature survey. (XLSX)

S6 Table The interaction of candidate miRNAs with genes associated with haloperidol drug function was predicted by miRWalk. (XLSX)

S7 Table In-silico target prediction of miR-22 and miR-29b with neuroscience panel of genes that were found to be altered with haloperidol treatment. (DOCX)
