# Supplementary material for: Haloperidol induces pharmacoepigenetic response by modulating miRNA expression, global DNA methylation and expression profiles of methylation maintenance genes and genes involved in neurotransmission in neuronal cells
Source: PLoS One. 2017 Sep 8;12(9):e0184209. doi: 10.1371/journal.pone.0184209 (PMC5590913; doi:10.1371/journal.pone.0184209)
Supplement: S2 Table — (DOCX) [file pone.0184209.s003.docx]

**Supplementary Table-2:** The table presents the mean fold changes in global DNA methylation relative to control at different haloperidol treatments and time intervals.

|  | 6 hr | 12 hr | 18 hr | 24 hr |
| --- | --- | --- | --- | --- |
| 1µM HLP | 0.94+0.23  (P=0.733) | 1.31+0.19  (P=0.097) | 1.26+0.10  (P=0.094) | 1.27+0.15  (P=0.093) |
| 10 µM HLP | 1.14+0.21  (P=0.379) | 1.52+0.27  (P=0.081) | 1.40+0.05  (P=0.071) | 1.47+0.04  (P=0.001) |
| 25 µM HLP | 0.92+0.06  (P=0.308) | 1.19+0.22  (P=0.271) | 1.13+0.05  (P=0.209) | 1.11+0.06  (P=0.096) |

P values are given in bracket.
