# Supplementary material for: Haloperidol induces pharmacoepigenetic response by modulating miRNA expression, global DNA methylation and expression profiles of methylation maintenance genes and genes involved in neurotransmission in neuronal cells
Source: PLoS One. 2017 Sep 8;12(9):e0184209. doi: 10.1371/journal.pone.0184209 (PMC5590913; doi:10.1371/journal.pone.0184209)
Supplement: S3 Table — (0 = not predicted as target by algorithm,1 = predicted as target by algorithm).The interaction of miR-29b with DNMT1,DNMT3A,DNMT3B and MBD2 was predicted by 2,7,7 and 4 algorithms respectively. (DOCX) [file pone.0184209.s004.docx]

**Supplementary Table 3**. Putative targets of miR-29b predicted by different algorithms in miRWalk. (0=not predicted as target by algorithm,1= predicted as target by algorithm).The interaction of miR-29b with *DNMT1*,*DNMT3A*,*DNMT3B* and *MBD2* was predicted by 2,7,7 and 4 algorithms respectively.

| *Algorithms* | *DNMT1* | *DNMT3A* | *DNMT3B* | *MBD2* |
| --- | --- | --- | --- | --- |
| *miRwalk* | 0 | 1 | 1 | 0 |
| *MicroT4* | 0 | 1 | 0 | 0 |
| *miRanda* | 0 | 1 | 1 | 1 |
| *miRDB* | 0 | 0 | 1 | 0 |
| *MiRMap* | 0 | 1 | 1 | 1 |
| *miRNAMap* | 0 | 1 | 0 | 0 |
| *Pictar2* | 0 | 0 | 1 | 0 |
| *PITA* | 1 | 0 | 0 | 0 |
| *RNAhybrid* | 1 | 1 | 1 | 1 |
| *Targetscan* | 0 | 1 | 1 | 1 |
| *SUM* | 2 | 7 | 7 | 4 |
