# Supplementary material for: Haloperidol induces pharmacoepigenetic response by modulating miRNA expression, global DNA methylation and expression profiles of methylation maintenance genes and genes involved in neurotransmission in neuronal cells
Source: PLoS One. 2017 Sep 8;12(9):e0184209. doi: 10.1371/journal.pone.0184209 (PMC5590913; doi:10.1371/journal.pone.0184209)
Supplement: S7 Table — (DOCX) [file pone.0184209.s008.docx]

**Supplementary Table 5**: In-silico target prediction of miR-22 and miR-29b with neuroscience panel of genes that were found to be altered with haloperidol drug treatment.

| **Gene** | **miR-22** | **miR-29b** |
| --- | --- | --- |
| **TSPO** | miRanda,PICTAR 5 |  |
| **DRD1** |  | DIANAmT,miRanda,miRwalk,RNAhybrid,PICTAR 5,Targetscan |
| **GCH1** |  | PICTAR5 |
| **ATP1A1** | miRWalk | miRanda |
| **ATP1B1** | RNA22 | DIANAmT,miRwalk,RNAhybrid,PICTAR 4,PICTAR 5,RNA 22,Targetscan |
